# Psychasthenia: Its Evolution, Course, Diagnosis, and Treatment

**Published:** 1907-10-19

**Authors:** Purves Stewart


### Notes on Current Neurology.

### PSYCHASTHENIA.

### Its Evolution, Course, Diagnosis, and Treatment.

By PURVES STEWART, M.D., F.R.C.P.

Psychasthenia may appear at any time of life, from childhood to old age; but the vast majority of cases show themselves in adolescence, at or shortly after the age of puberty. Psychasthenia occurs in subjects who are already abnormal—in impressionable, timid children, disinclined for exercise or sports, dreamy and introspective in nature. The earliest symptoms are often in the form of morbid scruples, which may take their origin in some absolutely trivial incident, such as small school-boy faults, the reading of some religious or "moral" book, some emotional shock, and so on. Whatever be the ostensible starting-point assigned by the young psychasthenic, the subsequent symptoms steadily evolve more or less rapidly. Gradually the bodily movements acquire a peculiar type, mental uncertainty and hesitation increase, the emotional reactions become excessive. With these there develop the imperious acts, all sorts of queer actions, such as over-frequent washing of the hands, constant aimless rummaging, a passion for verifying things of no importance, or a tendency to rumination and reverie. Last of all there come the obsessions and various forms of phobia, and the disease is now full-blown. It usually takes several years to reach the complete clinical picture, except when the onset is in adult life, in which case a few weeks may suffice for its development. Once established, the course of the disease is one of remissions. The psychasthenic patient has periods of relative calm, alternating with more or less acute "attacks" or paroxysmal periods.

During the intervals of calm, the fundamental symptoms of the disease, its "stigmata," can always be made out. The patients are feeble-willed and irresolute, incapable of sustained attention or intellectual effort; they are eccentric in manner and clumsy in all their acts, they tend to perform silly and useless movements, they acquire various tics, and finally develop a sort of panophobia or fear of everything strange or unexpected; they are also of a melancholy and hypochondriac disposition. These periods of calm may last for months or years. But, sooner or later, attacks or paroxysms are superadded, in which all the symptoms suddenly become accentuated, and some obsession or phobia looms so large as to occupy the patient's entire attention, to the exclusion of everything else. He becomes restless, flushed, more jerky than ever in his movements. Sometimes he is agitated, cannot remain still, and performs all sorts of gestures corresponding to his obsession. In other cases he is inert, listless, and apparently dull, mumbling indistinctly to himself. Some patients have marked vertigo. The attack lasts for a variable period, and then subsides, generally fairly quickly. These attacks may arise spontaneously without any apparent exciting cause; in other cases there are certain prodromal symptoms which are often recognised by the patient himself, and which may lead him to struggle against the approach of the paroxysm by various distractions such as talking, reading, physical exercise, hard work, and so on, but usually without avail. Some patients have a single obsession which recurs again and again in successive attacks; in other patients the obsessions are multiple, but one is predominant. In other cases, again, the obsession changes in different attacks.

Various clinical groups of psychasthenia can be recognised. The chief types, classified according to their obsessions, Raymond points out, are the following:—

1. Psychasthenics with obsessions of *doubt*, including the craving for hunting out objects, and the manias for counting or for naming things. This is the least dangerous variety, but at the same time the most persistent and tenacious.
2. Those with obsessions of *scruples*, in which everything is tinged with a morbid sense of moral right or wrong. Such patients are specially prone to self-mutilation.
3. Those with obsessions of *crime*. It is comparatively rare for this form of obsession to be translated into action, with the exception of kleptomania, to which the patients yield fairly easily.
4. Those with obsessions of *intoxication*, including dipsomania, morphinomania, etc., in which the impulse seems really irresistible.
5. The *sexual perverts*. Here also, like the preceding group of toxicomanias, and even in greater degree, the patient is liable to translate his obsession into action.
6. The so-called "*delirious*" type of psychasthenics. This is simply a delirious variety of any of the other types. The patient not only accuses himself of some form of crime, but does so to an exaggerated degree, and supports his self-accusations with the utmost ingenuity of argument.

Whatever be the variety of the disease, the patient, if left to himself, becomes steadily worse; he withdraws from social intercourse, and becomes in this respect a sort of ascetic. Delusions may develop, and he may drift into definite insanity of a melancholic type or with delusions of persecution. True dementia never occurs; the patients always remain capable of abstract reasoning. Other cases, if properly treated, may come to a standstill or even improve, so that the patient can lead an ordinary working life and mix in social intercourse. But the "stigmata" of the disease remain permanent.

As to the etiology of psychasthenia, all observers are agreed on the profound influence of heredity as by far the most important factor. Females are more liable than males. More than half the cases commence before the age of twenty. As to exciting causes, the most important are incidents which produce nervous exhaustion—such, for example, as emotional shock, mental over-work, traumatisms,

(especially those which are associated with profound emotional shock, *e.g.* railway accidents), influenza, enteric and other fevers. Freud, of Vienna, attributes an important *rôle* to genital troubles produced by sexual abstinence or by abnormal sexual irritation. Raymond, Janet, and the French school in general, however, do not agree with this view.

The diagnosis of psychasthenia is easy in well-marked cases. No other nervous malady has an analogous clinical picture. It is important to distinguish between the psychasthenic obsession and the normal "fixed idea" of the scientific worker or of the inventor. A normal fixed idea is in harmony with the regular course of thought; it is accepted and controlled by the will; it is practical in its object and independent of emotion of any kind. In all these respects it differs from the psychasthenic obsession. We must also distinguish between a psychasthenic obsession and a pathological "fixed idea" or delusion. A delusion is not recognised by the patient to be a delusion, nor is it in antagonism with his general tendencies; unlike an obsession it produces no mental struggle in the patient's mind, it develops in progressive fashion, and has nothing analogous to the acute "attacks" of the psychasthenic obsession.

Psychasthenia must also be distinguished from certain psychoses. Thus melancholia has a certain number of features in common with psychasthenia; for example, it has obsessions, scruples, attacks of acute distress, ideas of suicide, etc. But the melancholic patient has a fixed monotonous delusion, whereas the psychasthenic has a critical consciousness of his obsessions. The suicidal melancholic really desires death, whereas the suicidal psychasthenic is afraid that he may kill himself. Systematised delusions of persecution may, during the long initial stage of the disease, be confused with psychasthenia, but when the delusions are fully developed the diagnosis is comparatively simple. General paralysis of the insane may also have an initial stage not unlike psychasthenia. But when the characteristic physical signs appear, the diagnosis is easy. We have already pointed out the differences between psychasthenia and neurasthenia. Hysteria must also be clearly distinguished from psychasthenia. Doubled personality, as we have seen, is unconscious in hysteria; not so in psychasthenia. Hypnosis and suggestion, which constitute an integral part of hysteria, are impossible in pure psychasthenia.

The treatment of psychasthenia, as Janet observes, to be really efficacious ought to commence before birth; that is to say, people with a bad nervous heredity ought not to marry and have children. This, however, is too utopian, and in practice we have to deal with the patient as we find him. Treatment must be both moral and physical. As to moral treatment, or psychotherapeutics, this should consist in encouraging the patient to exercise his volition, and in pointing out to him the baselessness of his obsessions and phobiæ. Mere preaching at the patient, however, is of comparatively little value. We must recognise that most psychasthenics are physically below par and poorly nourished. Abundance of food, massage, cold baths, and physical exercises will all benefit the patient. Meanwhile the physician must familiarise himself with the patient's particular troubles and show that he understands them; he must try to show the patient that there is nothing extraordinary in his symptoms and thus afford the psychasthenic firm moral support. In most cases, removal of the patient from his old surroundings is necessary, placing him by preference in a special sanatorium, in the country if possible. Hypnotism, whatever its value in hysteria, is useless in psychasthenia. The moral influence of the physician is of supreme importance. He must encourage the patient and try to inspire him with faith in his ultimate cure.

